# Distance to sports facilities and low frequency of exercise and obesity: a cross-sectional study

**DOI:** 10.1186/s12889-022-14444-7

**Published:** 2022-11-07

**Authors:** Auriba Raza, Anna Pulakka, Linda L Magnusson Hanson, Hugo Westerlund, Jaana I. Halonen

**Affiliations:** 1 Department of Psychology, Stress Research Institute, Stockholm University, 106 91 Stockholm, Sweden; 2 Research Unit of Population Health, Faculty of Medicine, University of Oulu, P.O. Box 8000, 90014 Oulu, Finland; 3 Department of Public Health and Welfare, Finnish Institute for Health and Welfare, P.O. Box 30, 00271 Helsinki, Finland; 4 Department of Health Security, Finnish Institute for Health and Welfare, P.O. Box 30, 00271 Helsinki, Finland

**Keywords:** Proximity, Exercise, Physical inactivity, Physical activity facilities, Home, Work, Obesity

## Abstract

**Background:**

Little research has investigated the associations between proximity to physical activity facilities and behavior-related health and the majority have focused on proximity from home address. We add to the literature by examining proximity of these facilities to work and home address and including a wide range of physical activity facilities. We assess the associations for proximity of physical activity facilities from home and work address with self-reported frequency of exercise and obesity.

**Methods:**

Our analytical sample of 7358 participants was from the 2018 wave of the Swedish Longitudinal Occupational Survey of Health. We used logistic binomial regression adjusting for age, sex, education, civil status, individual socioeconomic status, neighborhood socioeconomic status, number of children under 12 years of age, work strain, and chronic disease.

**Results:**

Longer distance from home to paid outdoor and paid indoor physical activity facilities was associated with low frequency of exercise (fully adjusted Relative Risk for both 1.01, 95% CI 1.01–1.02). Associations of any or free outdoor facility with low frequency of exercise were not robust. Findings also indicated associations between long distance from workplace to any and paid outdoor facility and low frequency of exercise. Results for obesity were in the similar direction, however, these were not statistically significant.

**Conclusion:**

Increased distance of paid outdoor and paid indoor physical activity facilities from home and of paid outdoor facilities from work was associated with low frequency of exercise. Longitudinal and larger studies are needed to confirm our findings, particularly regarding obesity.

**Supplementary Information:**

The online version contains supplementary material available at 10.1186/s12889-022-14444-7.

## Introduction

It is estimated that physical inactivity accounts for six to ten percent of major non-communicable diseases worldwide [1, 2]. More than half of the European population does not meet physical activity levels recommended by World Health Organization [3, 4]. Labor saving devices, mechanization, and computerization have reduced physical activity both at home and work and the increased use of automobiles has reduced walking and bicycling for transportation [5]. To counter these decreases in physical activity and to improve public health, importance of activity promoting built environment has been recognized [5, 6].

Research on built environment and physical activity has focused on various environmental characteristics of the built environment such as street connectivity, residential density, availability of green space, cycle paths, sidewalks, access to recreational or exercise facilities, and neighborhood safety [7, 8]. Some studies have investigated a link between living in proximity to physical activity facilities and physical activity [9–16]. A six-country study observed positive associations between the number of private recreational facilities within 1 km and 0.5 km from home and the odds of engaging in non-walking leisure-time physical activity [11]. A Chinese study reported that individuals who lived within 10 min walking distance from an exercise facility were more likely to have any leisure time physical activity than those who lived further away [9]. Another study from China reported 6% decrease in recommended physical activity per 10 min increase in commuting distance to physical activity facility from home [16]. Moreover, a Finnish study reported average decrease physical activity among those whose distance to a sports facility increased compared to those for whom the distance remained close [12]. In an English city, those who lived within the closest quartile to a park were twice as likely to report five or more sessions of physical activity compared to those living in the farthest quartile. However, associations for proximity to sports center and gym with physical activity were not significant [10].

Prevalence of obesity has also increased worldwide and is contributing to the burden of non-communicable diseases [17]. Physical inactivity is a known risk factor for weight gain and thus, unavailability of sports facilities may also contribute to the epidemic of obesity. Studies on living in proximity to physical activity facilities and obesity or overweight are scarce. One study using UK biobank data reported that greater density of physical activity facilities within 1 km of home was independently associated with smaller waist circumference, lower body mass index (BMI), and body fat percentage [18]. Another study using perceived proximity measure associated presence of recreational facilities close to home with lower BMI [19].

The majority of existing research focuses on proximity of sports facilities to home address, while other contexts such as workplace, where individuals may spend much of their working days, could play a role in health and health behaviors [20]. Only few studies have looked at the associations between proximity of physical activity facilities to work and physical activity [14, 15] or obesity/overweight [19] and these have reported inconsistent findings. A study from West Scotland did not find associations between access to any, public, or private facilities within 800 m or 1600 m from work and physically active days [15]. A recent study from China, on the other hand, observed that short distance to physical activity facilities surrounding respondents’ work increased the amount of time spent on moderate and vigorous intensity physical activity [14]. Another study reported density of recreational facilities within one mile distance from work associated with lower BMI [19].

In Sweden, 68% of 15–74 years old are employed [21], workplace context, therefore, can be useful in engaging large majority of this population in physical activity and consequently improving public health. Thus, examining associations between access to physical activity facilities from work and behavior-related health in this population is relevant. Moreover, research using different facility types has been scarce although these may attract users differently and have a varying set of availability features (indoor vs. outdoor, paid vs. free). Thus, we have not been able to assess whether physical activity behaviors or BMI depend on the availability of any or different types of facilities. This study aims to determine association of proximity to physical activity facilities from home and work with low frequency of exercise and obesity. In addition to the inclusion of workplace context, the novelty of this study lies in the use of different categories of physical activity facilities; public and commercial as well as outdoor and indoor facilities and combinations of these.

## Materials and methods

### Study population

Our study population comprised participants of the Swedish Longitudinal Occupational Survey of Health (SLOSH) [22]. SLOSH cohort was established in 2006 with an aim to investigate associations of work environment factors with health. Originally, the SLOSH cohort consisted of respondents of the Swedish Work Environment Survey 2003 (SWES). In different years, the original SLOSH cohort was supplemented with additional participants from the first four biennial SWES waves [22]. SLOSH is an approximately representative sample of the Swedish active workforce aged 16–64 years at the time of the first response. Two different self-completed questionnaires have been sent to the SLOSH participants every second year since 2006; one for those who on average were gainfully employed (i.e. in paid work for ≥ 30% of full-time) during the three months prior to the survey, and another for non-employed (i.e. in paid work < 30% of full-time, or temporary or permanently unemployed).

For the current analyses, we used data from gainfully employed participants in the 2018 SLOSH wave (*N* = 10,386). We excluded participants with missing information on exposure, outcome, and any covariates leading to an analytic sample of 7358 participants. Descriptive characteristics of the included and excluded participants were similar (Supplementary Table 1).

The Swedish Ethical Review Authority has approved this study (Dnr: 2019–01,272).

### Exposure

Euclidean distances from participants’ home and work addresses at the end of year 2017 to the nearest sports facility were calculated by Statistics Sweden. Home addresses were from the Total Population Register [23] and work addresses from Statistics Sweden's Business Register [24]. Statistics Sweden aggregated different types of sports facilities into following categories:

*Sports hall facilities* included 846 swimming halls, 599 indoor ice rinks, 672 riding centers, 2741 school gymnasiums, and 46 multi-use arenas. The main source of the data was a property map dated on January 1^st^ 2019 obtained from Lantmäteriet (https://www.lantmateriet.se/sv/) [25]. Lantmäteriet is an authority belonging to the Ministry of Finance and it is responsible for the real estate division in Sweden and provides society with information on geography and real estates. Coverage of all included facilities has been improved by manually linking text information on the property map to unnamed building polygons by Statistics Sweden. Furthermore, all indoor ice rinks and riding centers have been confirmed using data from the Swedish Ice-hockey Federation and from individual riding centers’ website, respectively. All large school gymnasiums have been included, however, there were differences in how municipalities report school gymnasiums to Lantmäteriet. In some cases, individual municipalities have not reported small school gymnasiums to Lantmäteriet as they have been considered as an integral part of the school building, for example, and are thus not included in these data. All these indoor sports facilities were available for commercial use.

*Gyms and dance halls* include workplaces coded as fitness facilities (the Swedish Standard Industrial Classification code 93.130), and for cultural education (i.e., dancing, code 85.22). There were total of 871 gyms and 70 dancing related workplaces. These indoor facilities were also available for commercial use.

*Field sport facilities* include outdoor facilities such as ice-hockey rinks, speed-skating venues, bandy fields, football fields, track and field venues, basketball and baseball fields, and swimming pools from the property map. The property map has rather detailed information on these outdoor facilities which was manually assessed by Statistics Sweden. In total, 8962 sports facilities were included. These data have a good coverage and good inclusion rate except for basketball fields, baseball fields, and outdoor swimming pools, for which inclusion rate was lower. All these outdoor sports facilities were free for public use.

*Information on outdoor tennis courts* was also from the property map. Total of 1909 tennis courts were included and were available for commercial use.

*Golf courses and ski slopes* include total of 1015 facilities with almost complete coverage. The data were from Statistics Sweden for year 2015 and a handful of additional facilities were manually added from the property map for 2019. These outdoor facilities were available for commercial use.

*Outdoor sports tracks* include cross-country skiing areas and running tracks. Cross-country skiing tracks were retrieved for year 2019 from a Swedish webpage (www.skidspår.se) that maintains information about ski tracks and their maintenance and has very good coverage of all ski tracks in Sweden. Running tracks were derived from the property map and are based on municipal data reported to Lantmäteriet. The included running tracks are lit during evening time (except in the summer) and thus available for all-year use. In total, 1610 cross-country skiing tracks and 13,828 running tracks were included in this category and they were free for public use.

*Beaches* include European Union (EU) certified beaches and other public beaches. The data were obtained from the Swedish Agency for Marine and Water Management (HaV). Each municipality is obliged to provide information about EU-certified beaches for registry maintained by HaV. In addition, HaV encourages municipalities to also register other non-certified beaches. In total, this category includes 2476 beaches, including 438 EU-certified beaches. Beaches are free for public use.

We created five proximity variables reflecting distance to the nearest 1) any sport facility, 2) any outdoor, 3) free outdoor, 4) paid outdoor, and 5) (paid) indoor facilities. Outdoor facilities, including field sport facilities, outdoor tennis courts, golf courses and ski slopes, outdoor sports tracks and beaches, were further divided into free and paid facilities. Free outdoor sports facilities include field sport facilities, outdoor sports tracks, and beaches. Paid outdoor sport facilities include golf courses and ski slopes and outdoor tennis courts. Indoor sport facilities include sports hall facilities and gyms and dance halls, and all of these were considered as paid facilities. We additionally made variables for each category using distance to the nearest facility from either home or work.

### Outcomes

Frequency of exercise was estimated using a question “How much do you exercise? including walking and cycling to and from work” with response alternatives: i) exercise never, ii) move very little or take occasional walks, iii) exercise now and then, and iv) exercise regularly. The first or second response alternative were categorized as low frequency of exercise [26].

*Obesity* was based on BMI that was calculated as self-reported weight in kilograms (kg) divided by self-reported height in meters squared. Obesity was dichotomized as: Obese (30 ≤ BMI < 50) vs. not obese (14 ≤ BMI < 30).

### Covariates

Self-reported sociodemographic variables were occupational position (low, intermediate, high, and self-employed) and number of children (one or more children under 12 years). Information on age, sex, civil status (married/cohabiting vs. not), and educational attainment (secondary education or less, less than three years of tertiary education, and university education of three years or more) [27] was obtained from registers.

For home and workplace, neighborhood socioeconomic status (SES) variables, mean household income, low education (percentage of > 18 years old with only elementary school education) (values were inversed) and percentage of unemployment (values were inversed) of the population living within 500 m radius around participants’ home and workplace address were obtained from Statistics Sweden. For each determinant, standard z-scores (mean = 0, standard deviation = 1) were calculated. For both neighborhood SES variables, we calculated mean values across the three z-scores. Home and work neighborhood SES were divided into quartiles, the highest quartile indicating the highest neighborhood SES [20].

For chronic diseases, participants reported if they had hypertension, cardiovascular disease, diabetes, rheumatic disorders, and musculoskeletal disorders during the past two years. We created a dichotomized summary variable where value ‘0’ indicated no morbidity and value ‘1’ indicated having any one of these conditions [28]. Work strain was based on Swedish Demand Control Questionnaire [29]. We used five-job demand items (working fast, working hard, having excessive amount of work/too much effort, having enough time (reversed), having conflicting demands), while job control was based on six items (learning new things, needing high level of skills, being creative/initiative, having repetitive work, having a lot of say/what to do, having little freedom regarding how to do) [30]. Response options were; often = 1, sometimes = 2, rarely = 3 and never = 4. For each participant, mean values of the five-job demand and six job control items were calculated. The median of the means was used as a cut-off point. We categorized respondents as having work strain if their mean demand score was above the median and mean control score was below the median.

### Statistical analyses

We used logistic binomial regression (PROC GENMOD in SAS) to assess associations for distance to the nearest sports facility categories from home, work and home or work with low frequency of exercise and obesity. We first adjusted our models for age and sex (model 1). Model 2 was further adjusted for education, civil status, individual socioeconomic status, and home or workplace neighborhood socioeconomic status. In model 3, we added number of children under 12 years of age, work strain, and chronic disease.

As sensitivity analyses, sine the distance variables were skewed, natural logarithms were used to normalize the distributions of distances to the five sports facility categories for comparison purpose. Here, one-unit increase corresponds to doubling of distance, e.g., from 1 to 2 km.

All analyses were performed in SAS 9.4 (SAS Institute, Cary, North Carolina, USA) and results are reported as relative risks (RR) with 95% confidence interval (CI).

## Results

Table 1 presents characteristics of the study population. The participants had a mean age of 55 years (standard deviation 10), a majority (48%) held intermediate occupational positions, and slightly more than half (57%) were women. Prevalence of obesity (22%) was higher than that of low frequency of exercise (18%). Sports facilities were generally closer to workplace than home (Table 2).

**Table 1:** Descriptive statistics of the analytical sample of 7358 participants

Variable	N (%)
Outcomes (%)	
Low frequency of exercise	1306 (18)
Exercise never	162 (2)
Move very little or occasional walks	1144 (16)
Exercise now and then	2255 (31)
Exercise regularly	3707 (51)
Obese	1553 (22)
Co-variates (%)	
Age Mean (Standard Deviation)	54 (9)
Sex	
Women	4321 (59)
Presence of children under 12 years	1342 (18)
Civil status	
Cohabiting	5776 (79)
Education attainment	
Secondary education or less	2062 (28)
Less than three years of tertiary education	1661 (23)
University education of three years or more	3634 (49)
Occupational position	
Low	1907 (27)
Intermediate	3271 (46)
High	1908 (27)
Self-employed	61 (1)
Home Neighborhood SES	
Below median	3653 (50)
Above median	3705 (50)
Work Neighborhood SES	
Below median	3515 (51)
Above median	3413 (49)
Chronic disease	3308 (45)
Work strain	1442 (20)

**Table 2:** Descriptive statistics of the availability of types of sports facilities

	**Home (km)**				**Work (km)**				**Home or Work (km)**			
	N	Mean (SD)	Min	Max	N	Mean (SD)	Min	Max	N	Mean (SD)	Min	Max
Any Facility	7358	0.9 (1.2)	0	18.5	6939	0.5 (0.6)	0	11.3	7372	0.4 (0.5)	0	10.0
Any Outdoor	7358	1 (1.2)	0	18.7	6942	0.8 (0.7)	0	11.3	7372	0.6 (0.6)	0	13.1
Free Outdoor	7358	1 (1.2)	1	18.7	6939	0.8 (0.7)	0	11.3	7372	0.6 (0.5)	0	10.0
Paid Outdoor	7358	3 (3.7)	0.1	110	6942	2.2 (2.9)	0	111	7372	1.7 (2.6)	0	110
(Paid) Indoor	7358	2.4 (4.4)	0	83.7	6942	1.2 (3.3)	0	76.4	7372	1.0 (3.0)	0	76.4

Increase in distance from home to any, free outdoor, paid outdoor, and paid indoor facility was associated with an increased risk of low frequency of exercise in model 1, however, associations for any, free outdoor and paid indoor facility attenuated when adjusted for covariates (Table 3). Association between distance from home to paid outdoor sports facility was robust to further adjustments (fully adjusted RR 1.01, 95% CI 1.00–1.12).

**Table 3:** Associations between one kilometer increase in distance to the nearest sports facility type from home, from work, and from home or work with low frequency of exercise

	**Home**	**Work**	**Home or Work**
	RR (95% CI)	RR (95% CI)	RR (95% CI)
**Any Facility**			
Model 1 ^a^	**1.05 (1.00–1.10)**	**1.15 (1.05–1.26)**	**1.18 (1.05–1.33)**
Model 2 ^b^	1.02 (0.97–1.07)	1.07 (0.97–1.19)	1.09 (0.96–1.24)
Model 3^c^	1.02 (0.97–1.07)	1.07 (0.97–1.18)	1.09 (0.96–1.24)
**Any Outdoor**			
Model 1	1.04 (0.99–1.09)	**1.10 (1.00–1.20)**	1.11 (0.99–1.26)
Model 2	1.02 (0.97–1.07)	1.05 (0.96–1.15)	1.05 (0.92–1.20)
Model 3	1.01 (0.97–1.05)	1.03 (0.97–1.10)	1.04 (0.94–1.14)
**Free Outdoor**			
Model 1	**1.04 (1.00- 1.09)**	**1.10 (1.01–1.20)**	1.11 (0.99–1.24)
Model 2	1.02 (0.97–1.07)	1.05 (0.96–1.15)	1.04 (0.92–1.18)
Model 3	1.01 (0.98–1.05)	1.06 (0.99–1.12)	1.03 (0.94–1.13)
**Paid Outdoor**			
Model 1	**1.02 (1.01–1.04)**	**1.03 (1.01–1.05)**	**1.03 (1.01–1.06)**
Model 2	**1.02 (1.00–1.03)**	**1.02 (1.00–1.04)**	**1.02 (1.00–1.04)**
Model 3	**1.01 (1.00–1.02)**	**1.01 (1.00–1.03)**	1.01 (0.99–1.03)
**(Paid) Indoor**			
Model 1	**1.02 (1.01–1.03)**	1.01 (0.99–1.03)	1.01 (0.99–1.03)
Model 2	1.00 (1.00–1.02)	1.00 (0.98–1.02)	1.00 (0.97–1.02)
Model 3	**1.01 (1.00–1.02)**	1.00 (0.98–1.01)	1.00 (0.98–1.02)

^a^adjusted for age and sex

^b^adjusted for age, sex, education, civic status, individual socioeconomic status, and neighborhood socioeconomic status

^c^adjusted for age, sex, education, civic status, individual socioeconomic status, neighborhood socioeconomic status, number of children under 12 years of age, work strain, and chronic disease

There was indication of positive associations between increase in distance from workplace to any, any outdoor, free outdoor and paid outdoor facility and low frequency of exercise in model 1. While the effect estimates remained positive after further adjustments, they attenuated and did not reach statistical significance (fully adjusted RR 1.07, 95% 0.97–1.18 in relation to any facility, Table 3).

Increase in distance from either home or workplace to the nearest any and paid outdoor also indicated a positive association with low frequency of exercise in model 1. However, also these effect estimates attenuated after further adjustments, but they remained positive (e.g. fully adjusted RR 1.09, 95% CI 0.96–1.24 in relation to any facility) (Table 3).

When looking at obesity, the effect estimates were positive for associations between distance from home to any, any outdoor, free outdoor and paid outdoor facility, but were statistically significant only for model 1 (Table 4). There was no evidence for associations between distance from workplace to the different types of sports facilities.

**Table 4:** Associations between one kilometer increase in distance to the nearest sports facility type from home, from work, and from home or work and obesity

	**Home**	**Work**	**Home or Work**
	RR (95% CI)	RR (95% CI)	RR (95% CI)
**Any Facility**			
Model 1 ^a^	**1.06 (1.01–1.11)**	1.09 (0.99–1.19)	1.09 (0.97–1.23)
Model 2 ^b^	1.03 (0.98–1.08)	1.02 (0.93–1.13)	1.01 (0.89–1.15)
Model 3^c^	1.02 (0.97–1.07)	1.01 (0.92–1.12)	1.00 (0.88–1.14)
**Any Outdoor**			
Model 1	**1.06 (1.01–1.11)**	1.05 (0.96–1.15)	1.08 (0.97–1.22)
Model 2	1.04 (0.99–1.09)	1.02 (0.93–1.12)	1.05 (0.93–1.19)
Model 3	1.02 (0.98 -1.07)	1.01 (0.92–1.11)	1.03 (0.91–1.17)
**Free Outdoor**			
Model 1	**1.06 (1.01–1.10)**	1.07 (0.98–1.16)	1.11 (1.00–1.24)
Model 2	1.03 (0.99–1.08)	1.04 (0.95–1.13)	1.08 (0.96–1.21)
Model 3	1.02 (0.98–1.07)	1.02 (0.93–1.12)	1.06 (0.94–1.19)
**Paid Outdoor**			
Model 1	**1.02 (1.01–1.03)**	1.01 (0.99–1.03)	1.01 (0.99–1.03)
Model 2	1.01 (0.99–1.02)	1.00 (0.98–1.02)	1.00 (0.98–1.02)
Model 3	1.01 (0.99–1.02)	0.99 (0.97–1.01)	1.00 (0.97–1.02)
**(Paid) Indoor**			
Model 1	**1.01 (1.00–1.03)**	1.00 (0.98–1.02)	1.00 (0.98–1.02)
Model 2	1.01 (0.99–1.02)	0.99 (0.97–1.01)	0.98 (0.96–1.01)
Model 3	1.00 (0.99–1.02)	0.99 (0.97–1.01)	0.98 (0.96–1.01)

^a^adjusted for age and sex

^b^adjusted for age, sex, education, civic status, individual socioeconomic status, and neighborhood socioeconomic status

^c^adjusted for age, sex, education, civic status, individual socioeconomic status, neighborhood socioeconomic status, number of children under 12 years of age, work strain, and chronic disease

Results of the sensitivity analysis where logarithmic distances were used were similar to the main findings (Supplementary Tables 2 and 3). Estimates were stronger because these represent doubling in distance while non-logarithmic estimates represent 1 km increase in distance.

## Discussion

This study contributes to the existing literature on proximity of home to physical activity facilities and physical activity behavior and obesity, but also provides new aspects by examining associations for proximity of these facilities to work. Another major contribution is the use of a wide range of physical activity facilities and ability to categorize these into outdoor and indoor as well as paid and free facilities. We observed that longer distance from home to the nearest paid outdoor and paid indoor physical activity facility was associated with low frequency of exercise, and similarly, longer distance from work to the nearest paid outdoor physical activity facility demonstrated an increased risk of low frequency of exercise. For obesity, the effect estimates were positive, but not statistically significant, in relation to longer distance from home and from work to any facility, any outdoor, free outdoor, and paid outdoor.

Previous studies have reported associations between proximity of any sports or physical activity facilities to home and physical activity [9–16]. Few studies have investigated which category of facility, for example public or private, is of more importance to the study population [11, 13, 15]. Our results observed association between longer distance to paid outdoor physical activity facilities from home and increased risk of low frequency of exercise. Consistent with our findings, a study from San Diego conducted in 1990 reported association between greater density of paid facilities near home and three or more exercise sessions per week [13]. Similarly, a study from Scotland reported higher physical activity frequency where individuals lived closer to private facilities [15]. A large six-country study reported that individuals were more likely to engage in non-walking leisure time physical activity if they had a greater number of private recreational facilities within 0.5 or 1 km of the home [11]. Paid facilities and physical activity are usually positively associated with socioeconomic status, however, in our study associations remained significant after the effects of individual and neighborhood socioeconomic status were controlled for. A probable explanation could be that paid facilities are built in areas where demand for such services is high. Another explanation could be that individuals who visit paid facilities might have clearly defined patterns of physical activity and are thus likely to categorize themselves as physically active or regularly exercising [10].

The relationship between the availability of physical activity facilities and physical activity behavior cannot be completely understood without considering proximity of facilities to other locations such as workplace, where most adults spend majority of their waking time [11–13, 19, 31]. However, the handful of studies on associations between proximity of physical activity facilities to work and physical activity have not provided consistent findings [14, 15]. In our study, we observed significant associations between longer distance to paid outdoor facilities and low frequency of exercise, while, associations with any, any outdoor, and free outdoor facilities were significant when only adjusted for age and sex. Our results suggest physical activity promoting environment around workplace might contribute to positive health-related behaviors. However, larger, preferably longitudinal studies, are needed to confirm our findings and shed light on casual inference.

Physical activity facilities close to home and work provide convenient opportunities for physical activity. If improved access increases physical activity, one might expect to see a causal effect also on BMI [18]. Our results for distance to physical activity facilities from home and work and risk of obesity were in the similar direction as those for low frequency of exercise. In line with this, previous studies have reported positive associations of presence of physical activity facilities near home [18, 19] and work [19] with small waist circumference and low BMI.

From a policy perspective, our study provides some evidence that improved access can be associated with preferable physical activity behavior. However, there is a clear need for future studies to investigate the entwined roles of social and environmental factors in determining physical activity behavior. For example, people who are physically active or wish to be physically active may choose to live in areas with easy access to physical activity facilities. Along with availability of facilities, it might also be important to include individuals’ attitude towards physically active life style [10] that is likely to affect their activity levels.

There are some limitations to this study. Our frequency of exercise and obesity variables were based on self-reported data. People are likely to report health-behaviors in a positive direction which might introduce social desirability bias [32] and might have attenuated the observed associations. Secondly, our questionnaire did not have questions regarding the type or duration of exercise and thus is rather coarse measure of physical activity behavior. However, results for obesity were in the same direction as for low frequency of exercise which corroborate our findings for activity behavior. Another limitation is that for some people, workplace address may not be the actual location where they work, which may have caused some exposure misclassification. Lastly, due to the cross-sectional nature of the study design, causal inferences cannot be drawn; more physically active people may choose to live close to sports facilities, but it is unlikely that if a person reduces his/her exercise level the distance to sports facilities would change.

The major strength of our study was use of wide range of physical activity facilities which enabled us to categorize these into different types of facilities and investigate which category of facilities associates with low frequency of exercise and obesity. Another strength was that proximity to work was also examined since true relationship between proximity to physical activity facilities and low frequency of exercise and obesity may not be explained by limiting research to home context. We controlled for several possible individual level confounders but also home and workplace neighborhood SES.

## Conclusions

Increase in distance from home to paid outdoor and paid indoor physical activity facilities was associated with higher risk of low frequency of exercise. Increase in distance from work to paid outdoor physical activity was also associated with low frequency of exercise. However, associations with obesity were not clear.

## Supplementary Information

**Additional file 1:** **Supplementary Table 1.** Descriptive statistics of 3028 excluded participants. **Supplementary Table 2.** Associations between one unit increase in log-transformed distance to the nearest sports facility type from home, from work, and from home or work with low frequency of exercise. **Supplementary Table 3.** Associations between one unit increase in log-transformed distance to the nearest sports facility type from home, from work, and from home or work with obesity.

## Data Availability

The datasets generated and/or analysed during the current study are not publicly available due to ethical issues. However, we agree to allow the journal to review our data if requested, given that such a request can be granted by Stockholm University based on relevant legislation at the time of the request. Corresponding author should be contacted for the data requests.

## Abbreviations

BMI: Body Mass Index
CI: Confidence Interval
EU: European Union
HaV: Marine and water management
RR: Relative Risks
SES: Socioeconomic Status
SLOSH: Swedish Longitudinal Occupational Survey of Health
SWES: Swedish Work Environment Survey
