# Supplementary material for: Distance to sports facilities and low frequency of exercise and obesity: a cross-sectional study
Source: BMC Public Health. 2022 Nov 7;22:2036. doi: 10.1186/s12889-022-14444-7 (PMC9641919; doi:10.1186/s12889-022-14444-7)
Supplement: Supplementary file 1 — Additional file 1: Supplementary Table 1. Descriptive statistics of 3028 excluded participants. Supplementary Table 2. Associations between one unit increase in log-transformed distance to the nearest sports facility type from home, from work, and from home or work with low frequency of exercise. Supplementary Table 3. Associations between one unit increase in log-transformed distance to the nearest sports facility type from home, from work, and from home or work with obesity. [file 12889_2022_14444_MOESM1_ESM.docx]

**Distance to sports facilities and low frequency of exercise** **and obesity: a cross-sectional study**

**Supplementary Table 1. Descriptive statistics of 3028 excluded participants**

| Variable | N (%) |
| --- | --- |
|  |  |
| Outcomes (%) |  |
| Low frequency of exercise | 593 (20) |
| Exercise never | 74 (3) |
| Move very little or occasional walks | 519 (17) |
| Exercise now and then | 1019 (34) |
| Exercise regularly | 1367 (46) |
| Obese | 707 (24) |
| Co-variates (%) |  |
| Age Mean (Standard Deviation) |  |
| Sex |  |
| Women | 1728 (57) |
| Presence of children under 12 years | 811 (27) |
| Civil status |  |
| Cohabiting | 2356 (79) |
| Education attainment |  |
| Secondary education or less | 853 (28) |
| Less than three years of tertiary education | 735 (24) |
| University education of three years or more | 1434 (48) |
| Occupational position |  |
| Low | 819 (28) |
| Intermediate | 1357 (47) |
| High | 705 (24) |
| Self-employed | 29 (1) |
| Home Neighborhood SES |  |
| Below median | 1442 (48) |
| Above median | 1581 (52) |
| Work Neighborhood SES |  |
| Below median | 1389 (50) |
| Above median | 1403 (50) |
| Chronic disease | 1259 (42) |
| Work strain | 593 (20) |

**Supplementary Table 2**. Associations between one unit increase in log-transformed distance to the nearest sports facility type from home, from work, and from home or work with low frequency of exercise

|  | Home | Work | Home or Work |
| --- | --- | --- | --- |
|  | RR (95% CI) | RR (95% CI) | RR (95% CI) |
| Any Facility |  |  |  |
| Model 1 ^a^ | **1.15 (1.02-1.29)** | **1.38 (1.17-1.62)** | **1.45 (1.20-1.76)** |
| Model 2 ^b^ | 1.06 (0.94-1.20) | 1.18 (0.99-1.41) | 1.22 (0.99-1.51) |
| Model 3^c^ | 1.06 (0.94-1.19) | 1.17 (0.97-1.39) | 1.22 (0.98-1.50) |
|  |  |  |  |
| Any Outdoor |  |  |  |
| Model 1 | 1.11 (0.98-1.25) | **1.22 (1.02-1.46)** | 1.23 (0.99-1.53) |
| Model 2 | 1.05 (0.93-1.19) | 1.12 (0.94-1.34) | 1.10 (0.88-1.37) |
| Model 3 | 1.04 (0.92-1.18) | 1.10 (0.92-1.32) | 1.09 (0.87-1.36) |
|  |  |  |  |
| Free Outdoor |  |  |  |
| Model 1 | **1.13 (1.00-1.27)** | **1.22 (1.03-1.45)** | 1.20 (0.97-1.40) |
| Model 2 | 1.06 (0.94-1.19) | 1.12 (0.94-1.33) | 1.07 (0.86-1.32) |
| Model 3 | 1.03 (0.92-1.17) | 1.17 (0.98-1.39) | 1.06 (0.85-1.31) |
|  |  |  |  |
| Paid Outdoor |  |  |  |
| Model 1 | **1.14 (1.05-1.23)** | **1.14 (1.03-1.27)** | **1.15 (1.04-1.28)** |
| Model 2 | **1.08 (1.00-1.17)** | 1.08 (0.98-1.19) | 1.08 (0.97-1.20) |
| Model 3 | **1.08 (1.00-1.16)** | 1.08 (0.98-1.19) | 1.08 (0.97-1.20) |
|  |  |  |  |
| (Paid) Indoor |  |  |  |
| Model 1 | **1.01 (1.00-1.02)** | 1.00 (0.99-1.02) | **1.15 (1.05-1.27)** |
| Model 2 | **1.01 (1.00-1.02)** | 1.00 (0.98-1.01) | 1.03 (0.93-1.15) |
| Model 3 | **1.01 (1.00-1.01)** | 1.00 (0.98-1.01) | 1.04 (0.94-1.15) |
|  |  |  |  |

^a^ adjusted for age and sex

^b^ adjusted for age, sex, education, civic status, individual socioeconomic status, and neighborhood socioeconomic status

^c^ adjusted for age, sex, education, civic status, individual socioeconomic status, neighborhood socioeconomic status, number of children under 12 years of age, work strain, and chronic disease

**Supplementary Table 3**. Associations between one unit increase in log-transformed distance to the nearest sports facility type from home, from work, and from home or work with obesity

|  | Home | Work | Home or Work |
| --- | --- | --- | --- |
|  | RR (95% CI) | RR (95% CI) | RR (95% CI) |
| Any Facility |  |  |  |
| Model 1 ^a^ | **1.15 (1.02-1.29)** | **1.20 (1.03-1.41)** | 1.18 (0.97-1.44) |
| Model 2 ^b^ | 1.06 (0.95-1.18) | 1.05 (0.89-1.25) | 0.99 (0.80-1.24) |
| Model 3^c^ | 1.03 (0.93-1.15) | 1.03 (0.87-1.22) | 0.97 (0.79-1.21) |
|  |  |  |  |
| Any Outdoor |  |  |  |
| Model 1 | **1.15 (1.03-1.28)** | 1.10 (0.93-1.30) | 1.15 (0.94-1.41) |
| Model 2 | 1.07 (0.96-1.19) | 1.05 (0.89-1.24) | 1.09 (0.89-1.33) |
| Model 3 | 1.04 (0.94-1.16) | 1.02 (0.86-1.20) | 1.05 (0.86-1.28) |
|  |  |  |  |
| Free Outdoor |  |  |  |
| Model 1 | **1.14 (1.03-1.27)** | 1.13 (0.96-1.33) | 1.20 (0.99-1.45) |
| Model 2 | 1.07 (0.96-1.19) | 1.07 (0.91-1.26) | 1.12 (0.92-1.36) |
| Model 3 | 1.05 (0.99-1.16) | 1.04 (0.89-1.22) | 1.09 (0.90-1.32) |
|  |  |  |  |
| Paid Outdoor |  |  |  |
| Model 1 | **1.11 (1.05-1.19)** | 1.01 (0.92-1.10) | 1.04 (0.94-1.14) |
| Model 2 | 1.05 (0.98-1.12) | 0.96 (0.88-1.06) | 0.98 (0.89-1.08) |
| Model 3 | 1.03 (0.96-1.10) | 0.94 (0.86-1.03) | 0.97 (0.88-1.06) |
|  |  |  |  |
| (Paid) Indoor |  |  |  |
| Model 1 | **1.01 (1.00-1.02)** | 1.00 (0.99-1.01) | 1.05 (0.95-1.15) |
| Model 2 | 1.00 (0.99-1.01) | 0.99 (0.98-1.01) | 0.93 (0.84-1.04) |
| Model 3 | 1.00 (0.99-1.01) | 0.99 (0.98-1.00) | 0.94 (0.85-1.04) |
|  |  |  |  |

^a^ adjusted for age and sex

^b^ adjusted for age, sex, education, civic status, individual socioeconomic status, and neighborhood socioeconomic status

^c^ adjusted for age, sex, education, civic status, individual socioeconomic status, neighborhood socioeconomic status, number of children under 12 years of age, work strain, and chronic disease
